# Inhaled drug delivery: a randomized study in intubated patients with healthy lungs

**DOI:** 10.1186/s13613-023-01220-y

**Published:** 2023-12-11

**Authors:** Jonathan Dugernier, Déborah Le Pennec, Guillaume Maerckx, Laurine Allimonnier, Michel Hesse, Diego Castanares-Zapatero, Virginie Depoortere, Laurent Vecellio, Gregory Reychler, Jean-Bernard Michotte, Pierre Goffette, Marie-Agnes Docquier, Christian Raftopoulos, François Jamar, Pierre-François Laterre, Stephan Ehrmann, Xavier Wittebole

**Affiliations:** 1 Soins Intensifs, Cliniques Universitaires Saint-Luc, 1200 Brussels, Belgium; 2 Institut de Recherche Expérimentale et Clinique (IREC), Pôle de Pneumologie, ORL et Dermatologie, Université Catholique de Louvain, 1200 Brussels, Belgium; 3 Physiothérapie, Département des Thérapies, Hôpital Pourtales, Réseau Hospitalier Neuchâtelois, 2000 Neuchâtel, Switzerland; 4 Haute École Arc Santé, HES-SO, University of Applied Sciences and Arts of Western Switzerland, 2000 Neuchâtel, Switzerland; 5 Centre d’Etude des Pathologies Respiratoires, INSERM U1100, Faculté de médecine, Université de Tours, Tours, France; 6 Secteur de Kinésithérapie et Ergothérapie, Cliniques Universitaires Saint-Luc, 1200, Brussels, Belgium; 7 Médecine Nucléaire, Cliniques Universitaires Saint-Luc, 1200 Brussels, Belgium; 8 Pneumologie, Cliniques Universitaires Saint-Luc, 1200 Brussels, Belgium; 9 School of Health Sciences (HESAV), HES-SO, University of Applied Sciences and Arts of Western Switzerland, 1011 Lausanne, Switzerland; 10 Radiologie Interventionnelle, Cliniques Universitaires Saint-Luc, 1200 Brussels, Belgium; 11 Anesthésiologie, Cliniques Universitaires Saint-Luc, 1200 Brussels, Belgium; 12 Neurochirurgie, Cliniques Universitaires Saint-Luc, 1200 Brussels, Belgium; 13 CHRU Tours, Médecine Intensive Réanimation, CIC INSERM 1415, CRICS-TriggerSep F-CRIN Research Network, Tours, France; 14 Université de Tours, Tours, France; 15 Soins Intensifs, CHR Mons-Hainaut, 7000 Mons, Belgium

**Keywords:** Aerosol delivery, Invasive mechanical ventilation, Nebulizer

## Abstract

**Background:**

The administration technique for inhaled drug delivery during invasive ventilation remains debated. This study aimed to compare in vivo and in vitro the deposition of a radiolabeled aerosol generated through four configurations during invasive ventilation, including setups optimizing drug delivery.

**Methods:**

Thirty-one intubated postoperative neurosurgery patients with healthy lungs were randomly assigned to four configurations of aerosol delivery using a vibrating-mesh nebulizer and specific ventilator settings: (1) a specific circuit for aerosol therapy (SCAT) with the nebulizer placed at 30 cm of the wye, (2) a heated-humidified circuit switched off 30 min before the nebulization or (3) left on with the nebulizer at the inlet of the heated-humidifier, (4) a conventional circuit with the nebulizer placed between the heat and moisture exchanger filter and the endotracheal tube. Aerosol deposition was analyzed using planar scintigraphy.

**Results:**

A two to three times greater lung delivery was measured in the SCAT group, reaching 19.7% (14.0–24.5) of the nominal dose in comparison to the three other groups (*p* < 0.01). Around 50 to 60% of lung doses reached the outer region of both lungs in all groups. Drug doses in inner and outer lung regions were significantly increased in the SCAT group (*p* < 0.01), except for the outer right lung region in the fourth group due to preferential drug trickling from the endotracheal tube and the trachea to the right bronchi. Similar lung delivery was observed whether the heated humidifier was switched off or left on. Inhaled doses measured in vitro correlated with lung doses (*R* = 0.768, *p* < 0.001).

**Conclusion:**

Optimizing the administration technique enables a significant increase in inhaled drug delivery to the lungs, including peripheral airways. Before adapting mechanical ventilation, studies are required to continue this optimization and to assess its impact on drug delivery and patient outcome in comparison to more usual settings.

**Supplementary Information:**

The online version contains supplementary material available at 10.1186/s13613-023-01220-y.

## Background

The optimal administration technique to deliver aerosolized drugs to intubated patients with the highest drug dose penetrating the airways to the targeted lung parenchyma remains debated [1, 2]. Such technique is not standardized by international guidelines, resulting in variable and suboptimal practices reported in surveys in intensive care units (ICU) [3–5].

Mainly four configurations are suggested for aerosol therapy during mechanical ventilation (MV) when the nebulizer is operated continuously. Positioning the nebulizer far from the wye (30 cm of the wye [6–8] or at the inlet of the heated humidifier [9–12]) promotes the formation of an aerosol cloud within the inspiratory limb during expiration to deliver a bolus at the next insufflation increasing drug delivery in comparison to a closer position. However, positioning the nebulizer at 30 cm of the wye may require impractical circuit modifications with nebulizers. A theoretically optimized configuration was recently suggested with a ventilator circuit specifically designed for aerosol therapy (SCAT), i.e. pre-segmented inspiratory limb to position the nebulizer at 30 cm of a V-shaped wye, smooth inner surface and dry-inspired gas with the heat and moisture exchanger (HME) filter removed during the nebulization. This circuit was used in some patients in a recent randomized controlled trial for the prevention of ventilator-associated pneumonia with inhaled amikacin [13]. When a heated humidifier (HH) is required, the nebulizer is placed at the inlet of the humidification chamber with the HH switched off or left on. Switching off the HH was suggested based on previous studies reporting a higher inhaled dose with a conventional dry circuit in comparison to a heated humidified circuit [6, 14, 15]. The rational is to prevent aerosol hygroscopic growth and potential rainout that traps aerosol particles within the circuit and potentially prevents them to reach peripheral airways. However, authors recently questioned switching off the HH because of the residual condensation and heat within the circuit that prevents potential benefits. Moreover, potentially severe side effects may occur if the HH is not resumed at the end of the nebulization [16, 17]. Performing the nebulization with the HH left on is the third configuration supported in recent publications [1, 18]. The fourth configuration of aerosol delivery is to connect the nebulizer directly to the endotracheal tube (ETT) with the aim to combine the efficacy of drug delivery by reducing drug loss within the circuit during the inspiratory time (despite the expiratory losses) and feasibility as it could be applied with all types of circuits [19]. The four aforementioned configurations of aerosol delivery have not been compared in vivo, especially using specific ventilator settings minimizing air turbulences to improve lung delivery [20–22]. However, fixing specific settings to deliver an aerosol is questionable due to the risk of delayed weaning from MV [1].

Previous studies demonstrated that in vitro estimates of aerosol delivery can accurately reflect in vivo delivery during MV through either the comparison of the exhaled doses obtained with both methods [22] or the comparison of the inhaled doses measured in vitro and the drug concentrations in tracheobronchial secretions [6]. Direct in vitro*/*in vivo head-to-head comparisons are lacking. Radionuclide imaging methods are validated to assess lung deposition, including the regional distribution and penetration of inhaled aerosols into the lungs [23]. The aim of this study was to assess in vivo and in vitro the impact of a theoretically optimized configuration of aerosol delivery on intra- and extrapulmonary deposition of a radiolabeled aerosol administered using a vibrating-mesh nebulizer and specific ventilator settings in comparison to three other configurations in current use.

## Methods

### Design and patient selection

This randomized, comparative, double-blind study included postoperative neurosurgery ventilated patient. Eligibility criteria were a minimum age of 18 years and admission for brain neurosurgery or endovascular treatment of a brain aneurysm. Exclusion criteria were spine neurosurgery, history of cardiovascular and pulmonary disease. Patients were included if they had a healthy lung function defined as a ratio of the forced expiratory volume at 1 s to the forced vital capacity superior to 70%. Written informed consent was obtained from all participants before the surgery. The four configurations of aerosol delivery were randomized by a computer-generated random number list (Research Randomizer, Randomizer.org). XW generated the random allocation sequence while JD and GM enrolled the patients in the assigned configuration. The double-blind design was related to the patients and the data analysis. The study protocol was approved by the Institutional Medical Ethics Committee (B403201734204) and registered with ClinicalTrials.gov (NCT03464175, registered 13 March 2018, https://www.clinicaltrials.gov/study/NCT03464175).

### Nebulization procedure and invasive mechanical ventilation

**Fig. 1:**
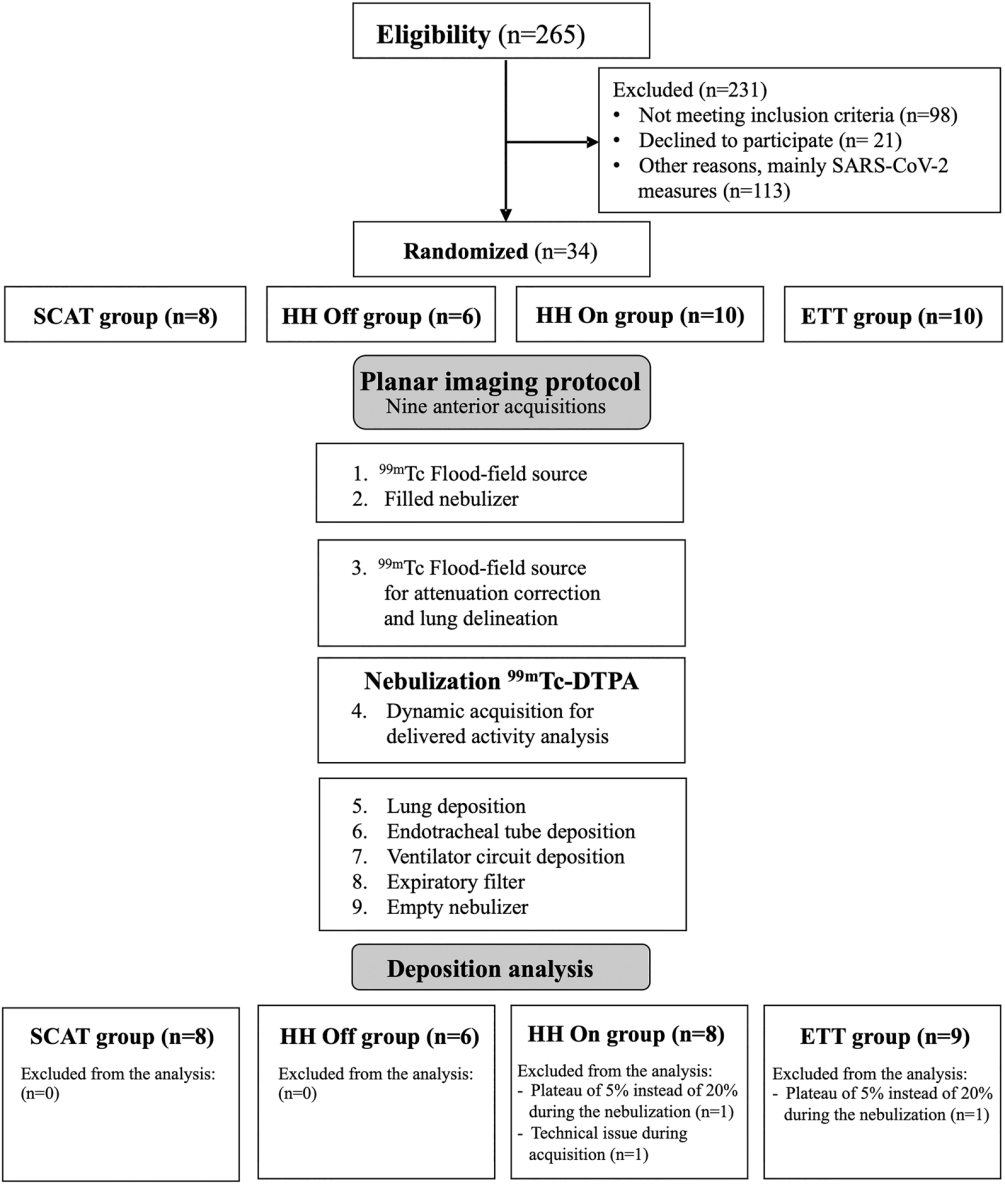
Summary of the protocol. ^*99m*^*Tc-DTPA* technetium-99 m labelled diethylenetriaminepentaacetic acid, *ETT* endotracheal tube, *HH* heated humidifier, *SARS-CoV-2* severe acute respiratory syndrome coronavirus 2, *SCAT* specific ventilator circuit for aerosol therapy

**Fig. 2:**
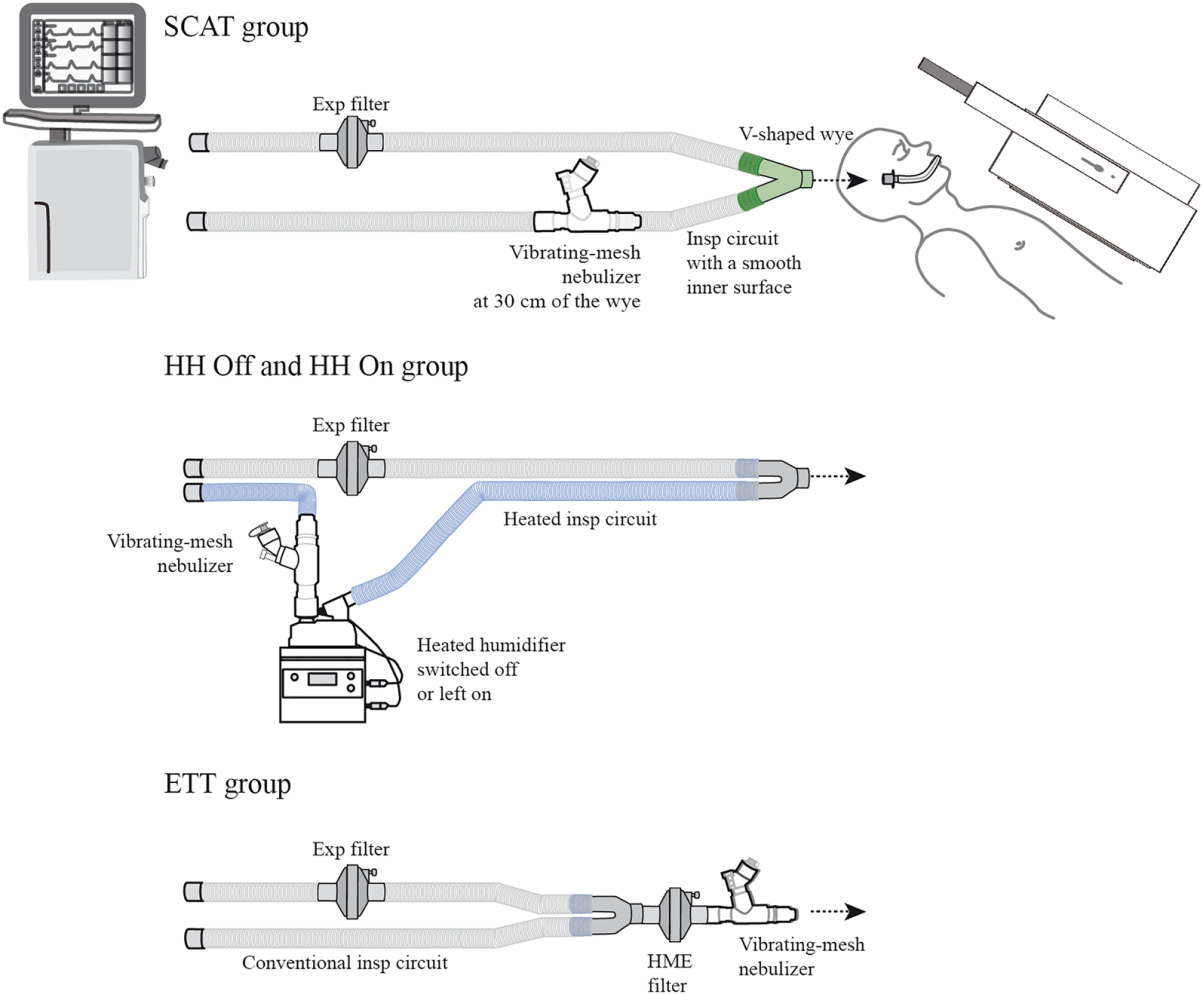
Illustration of the planar acquisition to assess aerosol lung deposition with four configurations of aerosol delivery. (1) A specific circuit for aerosol therapy (SCAT group) with the inspiratory limb pre-segmented at 30 cm of a wye to place the nebulizer, a smooth inner surface and a streamlined V-shaped wye (Reference 2154019, Intersurgical Ltd, Wokingham, UK) with the HME filter removed during the nebulization. (2) A conventional circuit equipped with a heated-humidifier (RT380, Fisher & Paykel Healthcare Ltd., Auckland, New Zealand) turned off 30 min before the nebulization (HH Off group) or (3) left on during the whole procedure (HH On group), with the nebulizer at the inlet of the HH. (4) A conventional circuit (IMMED, Brussels, Belgium) with the nebulizer directly connected between the endotracheal tube and an HME filter (ETT group). *ETT* endotracheal tube, *HH* heated humidifier, *SCAT* specific ventilator circuit for aerosol therapy

Aerosol particles were generated continuously by a vibrating-mesh nebulizer (Aerogen Solo®, Aerogen Ltd., Galway, Ireland). The nebulizer reservoir was filled with 3 mL technetium-99 m labelled diethylenetriaminepentaacetic acid (^99m^Tc-DTPA, 2 mCi). Patients were randomly assigned to four groups corresponding to four configurations of aerosol delivery during MV (Figs. 1 and 2). Patients were ventilated using an ICU ventilator (Servo U, Gentinge, Göteborg, Sweden) in volume control mode with theoretically optimized ventilator settings [20]: tidal volume of 8 mL/kg of ideal body weight, respiratory rate of 12 cycles/min, duty cycle (Ti/Ttot) of 50%, constant inspiratory flow pattern and an end-inspiratory pause of 20%. Positive end-expiratory pressure was set at 5 cmH_2_O. The bias flow was at 2 L/min. Sedative drugs (propofol infusion and a single dose of rocuronium) were injected to avoid patients’ movements, cough and patient-ventilator asynchrony.

### Image acquisition and deposition analysis

Image acquisitions were performed using a planar single detector gamma camera (Inter Medical Galaxy R, Inter Medical GmbH, Lübeck, Germany) to assess the pulmonary and extrapulmonary deposition (Fig. 1, detailed in Additional files 1, 2) [23, 24]. A penetration index was calculated as the outer-to-inner lung region ratio (O/I) from the ^99m^Tc-DTPA acquisition normalized to the O/I ratio from the ^99m^Tc flood-field acquisition [23]. A complementary analysis was performed to correct lung deposition data for drug trickling from the ETT and the tracheal area after the end of the nebulization if an increase of activity superior to 10% was measured in lung regions of interest. Pulmonary and extrapulmonary deposition of radiolabeled particles were expressed in counts or as a percentage of the nominal dose, i.e. the amount of radioactivity in the nebulizer at the beginning of experiments.

### In vitro–in vivo correlation analysis

All patient’s parameters were replicated in vitro using similar ventilator and settings, ventilator circuit and ETT. The same nebulizer used in vitro*–*in vivo, filled with the same solution (^99m^Tc-DTPA, 2 mCi/3 mL), were connected to an artificial lung model as described previously [7]. Inhaled doses, defined as the percentage of radiolabel deposited in a filter at the distal tip of the ETT and the extrapulmonary deposition were measured using a gamma camera (Orbiter 75 Ecam, Siemens Healthcare, Erlanger, Germany). Particle size analysis was assessed at the exit of the nebulizer by laser diffraction (Spraytec, Malvern Panalytical Ltd, UK) and at the distal tip of the ETT using a cascade impactor (IMAQ-GS-1E, California measurements Inc., Sierra Madre, CA).

### Statistical analysis

Statistical analysis was performed using SPSS software (version 29.0.0, IBM software). The estimated highest and lowest mean aerosol deposition values were 20% and 8%, respectively, based on previous data [10, 19]. Sample size calculation was based on a 5% standard deviation and a 7% expected difference in a mean deposition for a statistical power of 80%. No attrition rate was considered in the research design. Data are expressed as mean ± standard deviation (SD) or median [25–75% interquartile range (IQR)] depending on the data distribution normality test. The four-group comparison was conducted with a one-way analysis of variance (ANOVA). Both lungs were compared using a paired Wilcoxon test. Correlation between in vitro and in vivo measurements was conducted using the Pearson correlation coefficient (R). The intersubject variability of whole lung deposition was determined by the coefficient of variation. A *p*-value lower than 0.05 was considered significant.

## Results

Two hundred sixty-five patients scheduled for postoperative ICU admission after neurosurgery or endovascular treatment of brain aneurysm were screened for eligibility between March 2018 and September 2021 (Fig. 1). Among the thirty-four randomized patients, the first two patients were excluded because they were ventilated with an inspiratory plateau time of 5% instead of 20% during the nebulization. A third patient was excluded due to technical issues during scintigraphic acquisitions. The study included thirty-one patients (Table 1). The ETT diameter, the respiratory pattern, and the right to the left lung ventilation ratio (around 1.10) were comparable between the four groups (Table 2). Similar aerodynamic characteristics of aerosol particles were measured at the distal tip of the ETT for the four groups with a mean mass median aerodynamic diameter (MMAD) of 2.6 μm (Additional file 3).

**Table 1:** Patient characteristics

	SCAT group (*n* = 8)	HH Off group (*n* = 6)	HH On group (*n* = 8)	ETT group (*n* = 9)
Age (years)	52 ± 13	52 ± 17	52 ± 11	46 ± 8
Male, *n* (%)	4 (50)	3 (50)	2 (25)	3 (33.3)
Height (cm)	168 ± 9	172 ± 7	171 ± 6	172 ± 10
Body weight (kg)	74 ± 10	77 ± 12	75 ± 17	78 ± 13
Ideal body weight (kg)	63 ± 10	65 ± 9	63 ± 7	65 ± 10
Smoker, *n* (%)	3 (37.5)	0	1 (12.5)	3 (33.3)
Surgery, *n* (%)				
Brain tumor resection	3 (37.5)	2 (33.3)	5 (62.5)	6 (66.6)
Embolization of intracranial unruptured aneurysm	2 (25)	1 (16.6)	0	0
Neurosurgical clipping of an unruptured intracranial aneurysms	2 (25)	3 (50)	2 (25)	1 (11.1)
Stereotactic brain biopsy	0	0	1 (12.5)	1 (11.1)
Other (Arnold Chiary malformation, epileptic focus resection)	1 (12.5)	0	0	1 (11.1)
Lung function				
FEV_1_ (% predicted value)	94 ± 8	101 ± 13	92 ± 10	95 17
FVC (% predicted value)	93 ± 7	101 ± 8	93 ± 14	95 18
FEV_1_/FVC	83 ± 7	82 ± 4	82 ± 5	86 ± 5

Quantitative variables are expressed as mean ± SD. Qualitative variables are expressed as a proportion (%)

*ETT* endotracheal tube, *FEV*_*1*_ forced expiratory volume in 1 s, *FVC* forced vital capacity, *HH* heated humidifier, *SCAT* specific ventilator circuit for aerosol therapy

*p*-value > 0.05 for each group comparison

**Table 2:** Mechanical ventilation details and ventilatory pattern during inhalation

	SCAT group (*n* = 8)	HH Off group (*n* = 6)	HH On group (*n* = 8)	ETT group (*n* = 9)
ETT diameter (mm)	7.5 (7.0–8.5)	7.5 (7.0–8.0)	7.5 (7.0–8.0)	7.5 (7.0–8.0)
Right/left lung ventilation ratio	1.15 ± 0.13	1.11 ± 0.09	1.06 ± 0.16	1.15 ± 0.08
Ventilatory pattern during inhalation				
P_peak_ (cmH_2_O)	15 (15–17)	15 (15–16)	16 (15–17)	16 (15–17)
P_plat_ (cmH_2_O)	13 (12–15)	14 (13–14)	13 (13–15)	14 (12–15)
V_T insp_ (mL)	499 ± 60	518 ± 71	507 ± 54	520 ± 77
MV_insp_ (L/min)	6.01 ± 0.73	6.18 ± 0.83	6.08 ± 0.65	6.23 ± 0.91
Flow_peak insp_ (L/min)	20 ± 2	21 ± 3	20 ± 2	21 ± 3

The right/left lung ventilation ratio is the right to the left lung counts ratio obtained from the transmission scan using the ^99m^Tc flood-field source. Data expressed as mean ± SD or median (25–75% IQR)

*ETT* endotracheal tube, *HH* heated humidifier, *MV* minute ventilation, *P* pressure, *RR* respiratory rate, *SCAT* specific ventilator circuit for aerosol therapy, *V*_*T*_ tidal volume

*p*-value > 0.05 for each comparison

### Pulmonary deposition

**Fig. 3:**
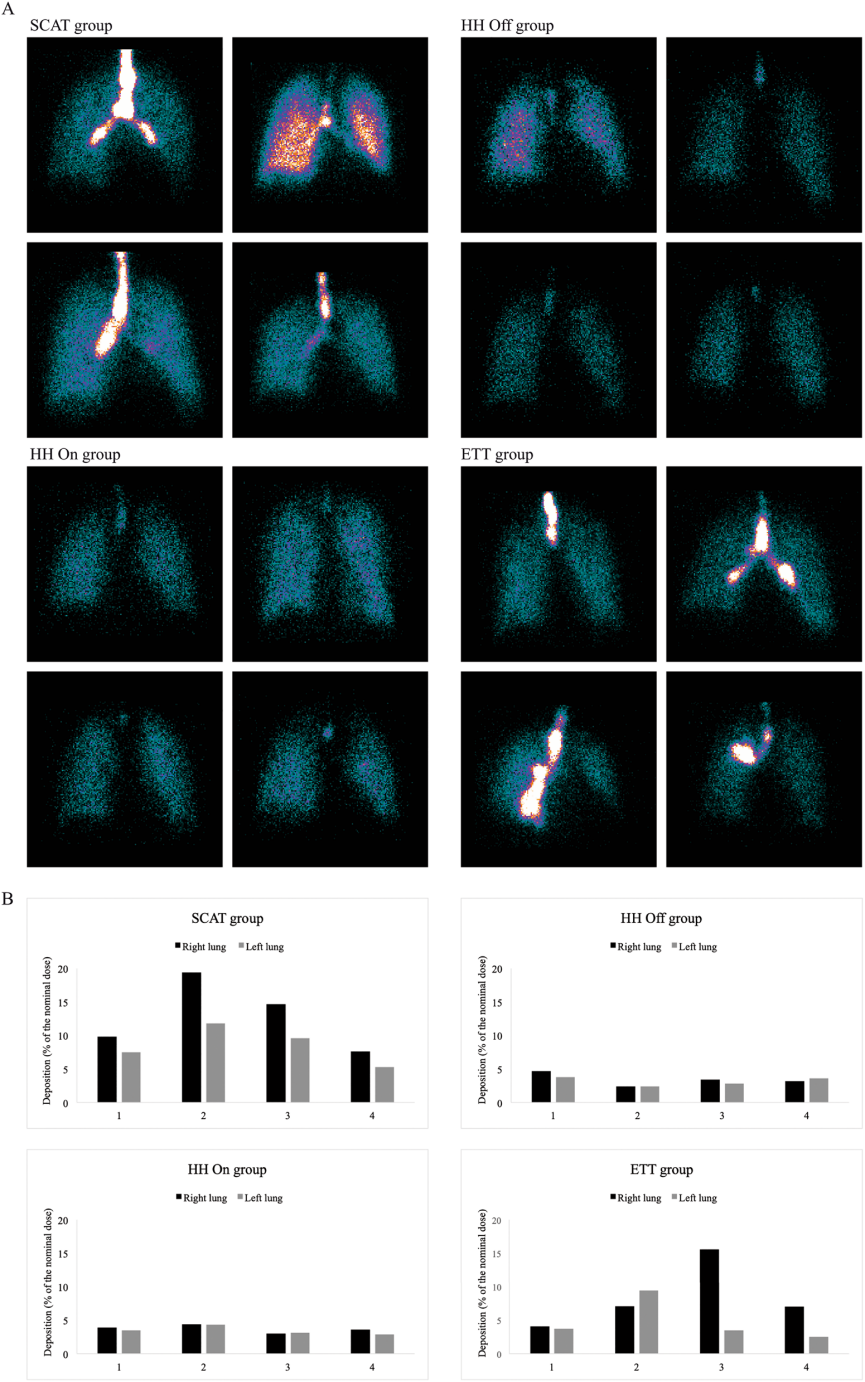
Intersubject variability of aerosol deposition between the right and the left lung and its penetration through the lungs with the four configurations of aerosol delivery. **A** Scintigraphic images of aerosol lung deposition in four patients of the SCAT group (*upper left*), the HH Off group (*upper right*), the HH On group (*lower left*) and the ETT group (*lower right*). Patients of the SCAT and the ETT group had a variable drug deposition (predominant right or left lung deposition or symmetrical) while a symmetrical aerosol deposition is depicted in the HH Off and the HH On group. **B** Radiolabeled drug dose deposited in the right and the left lung for the sixteen illustrated patients. Data expressed as a percentage of the nominal dose. *ETT* endotracheal tube, *HH* heated humidifier, *SCAT* specific ventilator circuit for aerosol therapy

Pulmonary and extrapulmonary deposition is detailed in Table 3. The amount of drug deposited into both lungs and into the right and the left lung analyzed separately, was significantly higher in the SCAT group (*p* < 0.01). Lung deposition was similar in the three other groups. From 50 to 65% of the lung deposition reached the outer lung regions while the rest was in the inner lung regions in all groups. Deposition in inner and outer lung regions was significantly increased in the SCAT group (*p* < 0.01), except for the outer right lung region in the ETT group. A similar penetration index of 0.7 was observed for both lungs in the HH groups. The penetration index was lower in the SCAT and the ETT group in comparison to the HH On group for the right lung (*p* < 0.05). The penetration index for the left lung was comparable in the four groups. A higher deposition into the ETT and the tracheal area was measured in the SCAT and the ETT group when compared to both HH groups (*p* < 0.001). When compared to the left lung, a higher right lung deposition was observed for the SCAT group only (*p* < 0.05). Intersubject variability of aerosol distribution between both lungs in each group is illustrated in Fig. 3A and B. The penetration index was comparable between both lungs in the four groups.

**Table 3:** Aerosol deposition in thirty-one postoperative neurological patients

	SCAT group (*n* = 8)	HH Off group (*n* = 6)	HH On group (*n* = 8)	ETT group (*n* = 9)
**Pulmonary deposition (%)**	19.7 (14.0–24.5)^*,†,‡^	6.2 (5.4–7.3)^§^	6.9 (6.6–7.4)^§^	9.2 (8.0–13.0)^§^
Right lung	10.6 (8.2–14.2)^*,†,‡^	3.1 (2.7–3.7)^§^	3.5 (3.0–3.9)^§^	4.7 (4.2–7.1)^§^
Inner region	5.2 (3.6–7.8)^*,†,‡^	1.2 (1.0–1.5)^§^	1.3 (1.2–1.5)^§^	2.2 (1.7–4.4)^§^
Outer region	5.0 (2.8–6.5)^*,†^	1.8 (1.7–2.3)^§^	2.2 (1.9–2.5)^§^	2.6 (2.3–3.0)
Penetration index	0.44 ± 0.12 (27)^*,†^	0.66 ± 0.10 (15)^§^	0.67 ± 0.07 (10)^‡,§^	0.47 ± 0.19 (40)^†^
Left lung	9.1 (6.6–10.3)^*,†,‡^	3.1 (2.6–3.7)^§^	3.5 (3.1–4.1)^§^	3.8 (3.3–4.9)^§^
Inner region	3.9 (2.7–4.3)^*,†,‡^	1.1 (0.9–1.3)^§^	1.3 (1.2–1.4)^§^	1.4 (1.2–2.0)^§^
Outer region	5.2 (3.6–6.3)^*,†,‡^	2.0 (1.7–2.3)^§^	2.1 (1.9–2.7)^§^	2.3 (2.0–2.9)^§^
Penetration index	0.56 ± 0.17 (30)	0.73 ± 0.10 (14)	0.69 ± 0.11 (16)	0.57 ± 0.13 (23)
Right/left lung ratio	1.31 ± 0.23 (17)	1.04 ± 0.17 (16)	1.01 ± 0.19 (19)	1.66 ± 1.23 (74)
**Extrapulm. deposition (%)**	80.3 (75.5–86.0)^*,†,‡^	93.8 (92.7–94.6)^§^	93.1 (92.5–93.4)^§^	90.8 (87.0–92.1)^§^
ETT and Tracheal area	23.9 (20.3–26.4)^*,†^	11.9 (11.1–12.1)^‡,§^	7.4 (6.7–8.6)^‡,§^	19.2 (16.7–24.0)^*,†^
Ventilator circuit	50.5 (48.1–56.3)^*,†,‡^	78.2 (76.8–79.0)^‡,§^	81.5 (79.7–81.7)^‡,§^	65.5 (58.3–71.2)^*,†,§^
Inspiratory circuit	38.6 ± 5.5 (14)^*,†,‡^	68.9 ± 2.9 (4)^‡,§^	73.7 ± 2.9 (4)^‡,§^	13.5 ± 5.7 (42)^*,†,§^
Inspiratory limb	27.0 ± 9.1 (34)^†^	31.2 ± 2.5 (8)	34.7 ± 3.0 (9)^§^	–
Humidification chamber	–	31.3 ± 3.4 (11)	32.1 ± 5.6 (17)	–
Nebulizer T-piece	11.6 ± 5.0 (43)	6.4 ± 1.4 (21)^‡^	6.9 ± 1.9 (27)^‡^	13.5 ± 5.7 (42)^*,†^
Expiratory circuit	13.1 (11.0–15.5)^†,‡^	9.1 (7.0–11.2)^‡^	6.3 (6.0–8.4)^‡,§^	52.1 (46.5–57.0)^*,†,§^
Nebulizer retention	4.6 ± 0.11 (2)	4.2 ± 0.09 (2)	4.5 ± 1.3 (28)	4.3 ± 0.13 (3)

The penetration index was the outer to inner lung region ratio (O/I) from the ^99m^Tc-DTPA acquisition normalized to the lung volume, i.e. divided by the O/I ratio from the ^99m^Tc flood-field acquisition. A penetration index inferior to 1 indicated a major deposition in the inner region, mainly composed of central airways. Deposition in the expiratory circuit included the drug dose deposited in the expiratory limb and exhaled in the expiratory filter, including the heat and moisture exchanger filter in the ETT group. Data expressed as mean ± SD (coefficient of variation, %) or median (25–75% IQR) percentage of the nominal dose

*ETT* endotracheal tube, *HH* heated humidifier, *SCAT* specific ventilator circuit for aerosol therapy

^*^ *p* < 0.05 vs HH Off group; ^†^*p* < 0.05 vs HH On group; ^‡^*p* < 0.05 vs ETT group; ^§^*p* < 0.05 vs SCAT group

**Fig. 4:**
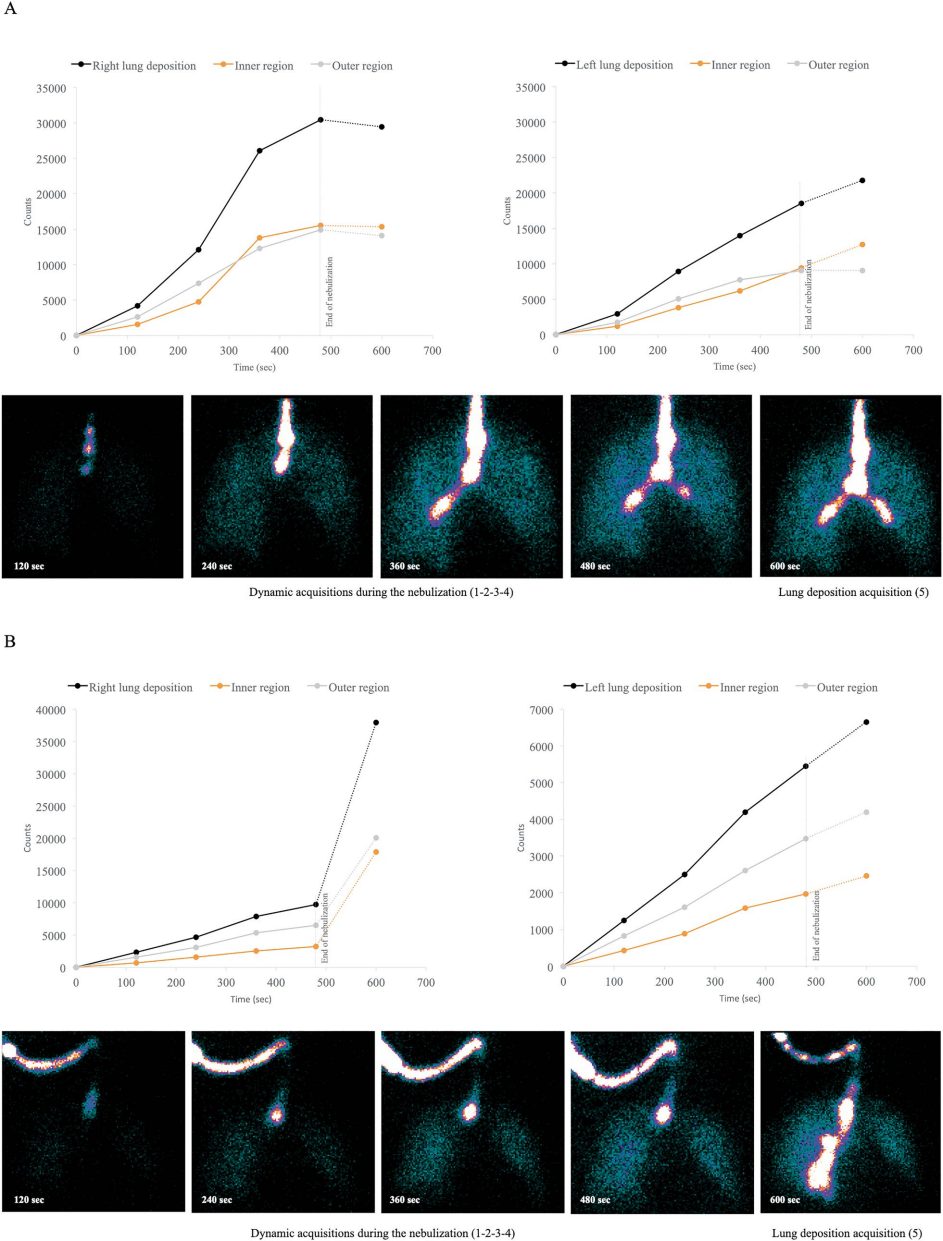
Illustration of drug trickling from the endotracheal tube to the lungs in a patient from (**A**) the SCAT group and **B** from the ETT group. Graphics depict the evolution of activity deposited in lung regions for the right and the left lung throughout the dynamic acquisition during the nebulization (plain line) and after the nebulization (dashed line). Data point 4 of shorter acquisition duration was normalized to the 120-s acquisitions of other data points. *Patient A* Drug trickling was observed during the nebulization as indicated by the exponential increase of counts in the right inner region and after the nebulization in the left lung as indicated by the increase of activity inside the inner region while the activity inside the outer lung region plateaued. *Patient B* Drug trickling was observed in both lungs, especially in the inner to the outer right lung region. *ETT* endotracheal tube, *SCAT* specific ventilator circuit for aerosol therapy

Interestingly, the dynamic acquisition revealed a further increase of the activity in lung regions after the nebulization in three patients from the SCAT group and three patients from the ETT group (Additional file 4): this phenomenon likely represents drug trickling from the ETT to the lungs as depicted in Fig. 4. Deposition data were corrected for drug trickling from the ETT to the lungs during the 2-min acquisition for lung deposition after the nebulization (Additional file 5). The difference was not significant whether drug trickling was accounted for or not in each group. However, high drug trickling in the outer right lung regions observed in the ETT group explained the similar deposition in the outer right lung regions measured in the SCAT and the ETT group before the correction for this phenomenon.

### Extrapulmonary deposition

Aerosol loss within the ventilator circuit was lower in the SCAT group (50.5% of the nominal dose) in comparison to the three other groups (65.5 to 81.5% of the nominal dose). More than 60% of the aerosol was trapped within the circuit whether the HH was switched off or left on. In the ETT group, 50% of the nominal dose was lost within the HME filter and the expiratory circuit. Residual doses in the nebulizer reservoir were around 4% of the nominal dose. Nebulization lasted 6 ± 2 min. No radioactivity was detected on the expiratory valve. No ambient and surface contamination was detected after the procedure.

### In vitro–in vivo correlation

**Fig. 5:**
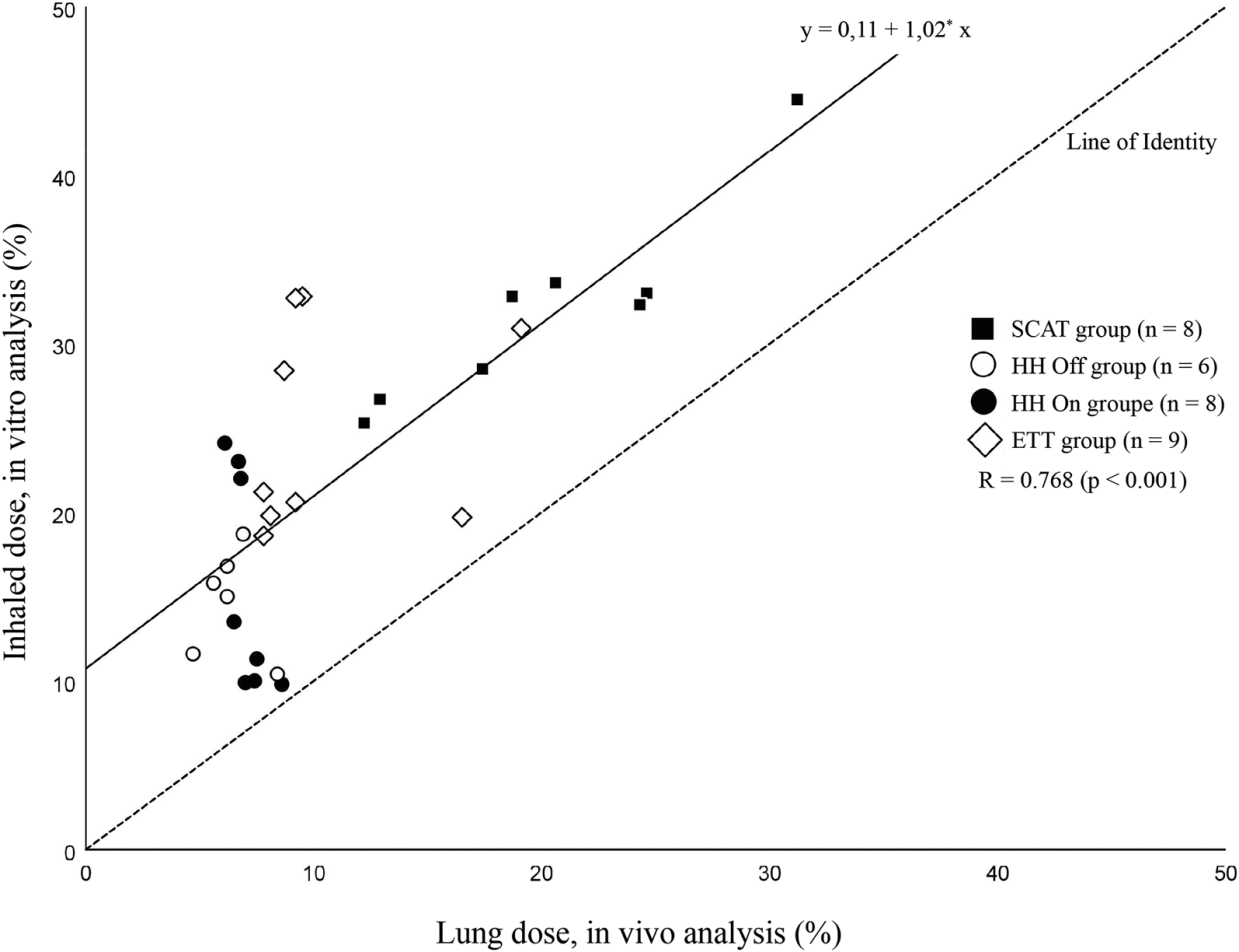
Correlation between inhaled doses of radiolabel measured in vitro in a filter positioned at the distal tip of the endotracheal tube and lung deposition of radiolabel measured in vivo using planar scintigraphy. Data are expressed as a percentage of the nominal dose. A correlation was found for the whole dataset (*R* = 0.768, *p* < 0.001). The dashed line is the line of identity and the plain line is the regression line with the corresponding equation. *ETT* endotracheal tube, *HH* heated humidifier, *SCAT* specific ventilator circuit for aerosol therapy

Inhaled doses and the extrapulmonary deposition resulting from the in vitro replication of the thirty-one patients are detailed in Additional file 6. A significant correlation was found between the inhaled doses measured in vitro and lung deposition measured in vivo for the whole data set (*R* = 0.768, *p* < 0.001). However, a subgroup analysis reported a significant correlation between in vitro and in vivo data for the SCAT group only (*R* = 0.925, *p* < 0.001), not for the other groups. Inhaled doses overestimated lung deposition (around 10%) in all patients (Fig. 5).

## Discussion

This is the largest in vitro-in vivo clinical scintigraphic study analyzing drug delivery using specific ventilator settings with the three configurations of aerosol delivery in current use for aerosol therapy compared to a theoretically optimized configuration.

The optimized SCAT configuration improved drug delivery with 20% of the nominal dose deposited in the lungs, two to three times the doses measured with the three other configurations. Considering the continuous nebulization, placing the nebulizer at 30 cm of a streamlined V-shaped wye in a dry circuit with a smooth inner surface reduced aerosol loss in the ventilator circuit (50%) in comparison to the same nebulizer positioned farther from the patient, at the inlet of the HH (80% lost mainly in the inspiratory circuit), or connected to the ETT (65% lost mainly in the HME filter during expiration). Ventilator settings were not influential factors in aerosol deposition between groups since similar settings were used for all patients. The benefit of using dry gas on lung deposition in the SCAT group could not be isolated from other factors enhancing drug delivery in this group related to the position of the nebulizer and the characteristics of the circuit (smooth inner surface, V-shaped wye). There is no benefit to switching off the HH 30 min before the nebulization (i.e. maximal duration for safety reasons [25]) neither to increase the lung doses nor to enhance drug penetration through the airways in comparison to the HH left on. Considering the absence of benefit and the potential damage to the bronchial mucosa if the HH is not resumed at the end of the nebulization, this practice should not be recommended.

We observed greater lung doses in comparison to previous in vivo scintigraphic studies (3 to 15% of the nominal dose) [26]. However, these remain low considering the inhaled doses of 37% and 72% reported with breath-actuated jet [27] and vibrating-mesh nebulizers [28], respectively. Several factors impaired lung delivery related to the continuous nebulization such as the characteristics of the circuit, the nebulizer position and the bias flow. Depending on the bias flow, positioning the nebulizer far from the wye increases drug delivery [2, 6, 9]. However, this study shows that the circuit acts as a trap more than a spacer, especially when the nebulizer is placed at the inlet of the HH with a bias flow of 2 L/min. Imposing a higher bias flow with another brand of ventilator would have probably increased lung delivery with the HH configuration, as suggested in vitro [9, 12]. Further studies are needed to recommend an efficient and feasible technique for all intubated patients, which could be applied to all brands of ventilators. Using an efficient breath-actuated nebulizer placed closer to the patient may fix the problem of ventilator compatibility (bias flow, circuit, humidification) and may question the necessity to adapt the ventilator settings using sedatives, as suggested previously [27]. Before promoting the SCAT configuration, its superiority over more usual configurations should be confirmed using other ventilator settings and bias flow. The SCAT circuit is to date not commercially available and adding a 30-cm tubing extension to reproduce this configuration should not be recommended.

This study confirms that inhaled drugs may reach the lung periphery of intubated patients as around 50 to 65% of lung deposition reaches the outer lung regions mainly composed of peripheral airways. However, the outer region involves more lung tissue than the inner region [23]. Normalized to the volume of tissue, we found that most inhaled drug was limited to the inner region mainly composed of central airways, as indicated by a penetration index below 1. Previous scintigraphic studies also reported a penetration index between 0.4 and 0.7 [19, 29]. Nonetheless, such penetration index in intubated patients is equivalent to spontaneously breathing healthy subjects [30]. We measured particles around 2.6 μm reaching the trachea which is more likely deposited within the central airways [31]. As recently suggested by Usmani et al., generating extra-fine particles inferior to 2 μm may help to increase peripheral airways deposition [32]. Interestingly, our results characterize the particle distribution emitted by the nebulizer (MMAD around 3.4 µm). While larger particles were filtered in the circuit in the HH groups, they deposited in the proximal pieces, the ETT, the trachea and the first bronchi in the SCAT and the ETT group (see Fig. 3). Drug trickling from the ETT to the lungs only impacted the latter groups. We quantified drug trickling only after the nebulization, but it also occurred during the nebulization (patient A in Fig. 4). It tended to increase lung doses and drug penetration from central to peripheral airways with a preferential deposition towards the right bronchi (patient B in Fig. 4). It may also explain the great intersubject variability of lung deposition observed in both groups and previously [19]. In addition, deposition by drug trickling might also explain the high antibiotic concentrations measured in tracheobronchial secretions samples and the contamination of the epithelial lining fluid measured in patients with ventilator-associated pneumonia [33, 34].

The correlation between in vitro and in vivo data confirmed the importance of bench studies to develop the technique. Estimating lung deposition from inhaled doses obtained in vitro might require, however, higher delivered doses. Our subgroup analysis revealed the absence of correlation between in vitro and in vivo data for the three configurations with lower drug delivery (ETT and HH groups). Lower inhaled doses measured for the in vitro replicates of the HH and the ETT groups were impacted by higher variability in comparison to the lung doses measured in vivo in the same groups. Potential explanations are the variation in exhaled doses and drug trickling from the ETT included in inhaled doses [22] and the intradevice variability of delivery reported previously with the nebulizers used in this study [35].

The impact of specific ventilator settings on drug delivery, regional deposition, and patient outcome in comparison to more usual settings for critically ill patients merits further evaluation before altering the management of MV. Indeed, previous studies testing the same nebulizer in similar configurations measured higher inhaled doses [9, 11] and similar penetration index [19] with more usual ventilator settings.

This study has limitations. Pulmonary deposition of a radiotracer cannot be directly extrapolated to the deposition of a specific drug in patients with lung diseases. We included patients with healthy lungs to assess the impact of the inhalation technique without any influences of lung diseases. Second, ventilator settings were fixed for all patients using sedatives to theoretically optimize lung deposition. However, lung deposition data obtained in this study would have been very likely different with ventilator settings adapted to the patient’s need (e.g. lower tidal volume, lower duty cycle or spontaneously breathing activity in pressure support ventilation). Third, the penetration index was based on a two-dimensional analysis of lung delivery overestimating central airways deposition due to the overlap of peripheral airways in the inner region of interest [36]. Tomographic studies are needed for a more precise evaluation of peripheral airways deposition. Fourth, counts for quantification were not based on a geometric mean from anterior and posterior images but on a single anterior planar image due to bed restriction. It could decrease the accuracy of the quantification. This potential bias was, however, constant for all patients. Fifth, 75% of the vibrating-mesh nebulizers used in this study were interrupted during nebulization in both in vitro and in vivo* experiments* due to bubbles between the mesh and the solution preventing it from being aerosolized (1 to 3 interruptions in most runs, till 5 interruptions in one run only). This issue may affect the aerosol generation and prolong the nebulization [37]. Investigators resolved it immediately as the nebulization was not prolonged, except for two patients (14-min duration). Nebulization ran completely to obtain similar residual doses (4–6%) in all groups. However, clinicians should be aware of the potential risk of interruption of treatment, as reported by Block et al. [38]. This issue could be easily resolved by gentle agitation, dabbing the reservoir or the replacement of the nebulizer.

In conclusion, this study demonstrated the difficulty to reach high drug doses in the lungs of intubated patients using current administration techniques and the challenge to standardize the technique. Lung doses below 10% of the nominal dose were measured with the three administration techniques in current use. There is no interest to turn off the heated humidifier during the nebulization to improve drug delivery. The SCAT challenging technique developed herein allows lung doses of 20%, with 55% reaching the outer lung regions. However, it requires specific ventilator settings, bias flow and circuit adaptations. Lung doses and drug penetration measured in patients with healthy lungs in this comparative trial might not reflect inhaled drug delivery in critically ill patients with lung diseases (e.g. bronchoconstriction, tracheobronchial secretions or alveolar infiltrates). Considering low lung deposition, adapting the nominal drug dose might be a therapeutic option for further clinical studies. In vitro design should be continued to define an efficient and feasible technique as it correlates with lung deposition, even if it overestimates it. Further tomographic studies are needed to test it in vivo*,* contributing to defining drug doses in pathological central and peripheral airways to elaborate clinical studies.

### Supplementary Information

**Additional file 1.** Additional details on methods.

**Additional file 2.** Definition of lung regions to assess aerosol distribution and penetration in the right and the left lung during invasive mechanical ventilation. (A) Both lungs were divided into inner (I) and outer (O) region of interest using the ^99m^Tc flood ventilation scan. The limits of the rectangular outer lung region were defined as the 36% isocount contour of peak counts in the lung field without artefacts (i.e. “islands” or “peninsulas” inside or outside of the lung field). The inner region was automatically designed as half of the width of the outer region and one-half of its height. (B) Regions of interest were copied on the 99mTc-DTPA deposition scan to quantify the inhaled radiolabeled drug deposition.

**Additional file 3.** Aerodynamic characteristics of aerosol particles.

**Additional file 4.** Evolution of the activity deposited inside the right and the left lung regions was measured throughout the dynamic acquisition during the nebulization (plain line) and after the nebulization (dashed line). All data points were normalized to 120-s acquisitions. Three patients from the SCAT group (asterisk) and three patients from the ETT group (asterisk) had an increase of counts superior to 10% between the last dynamic acquisition and the acquisition for lung deposition, a difference considered significant as it exceeds the Poisson error inherent to planar scintigraphy analysis. This increase in activity after the nebulization was linked to drug trickling from the endotracheal tube and the tracheal area to the lungs. This phenomenon probably happened even during the nebulization, as indicated by an exponential increase of counts, but cannot be quantified. ETT, endotracheal tube; HH, heated humidifier; SCAT, specific ventilator circuit for aerosol therapy.

**Additional file 5.** Aerosol deposition after correction for drug trickling from the endotracheal tube to the lungs after the nebulization.

**Additional file 6.** In vitro analysis of aerosol deposition in invasive mechanical ventilation.

## Data Availability

The datasets used and analyzed during the current study are available from the corresponding author upon reasonable request.

## Abbreviations

^99m^Tc-DTPA, 2 mCi: Technetium-99 m labelled diethylenetriaminepentaacetic acid
ETT: Endotracheal tube
HME: Heat and moisture exchanger filter
ICU: Intensive care unit
IQR: Interquartile range
MV: Invasive mechanical ventilation
HH: Heated humidifier
MMAD: Mass median aerodynamic diameter
O/I: Outer-to-inner lung region ratio
R: Pearson correlation coefficient
SCAT: Specific circuit for aerosol therapy
SD: Standard deviation

## Declarations

### Prior abstract presentation/publication

The abstract was not presented or published in any form.
