# Supplementary material for: Inhaled drug delivery: a randomized study in intubated patients with healthy lungs
Source: Ann Intensive Care. 2023 Dec 11;13:125. doi: 10.1186/s13613-023-01220-y (PMC10710976; doi:10.1186/s13613-023-01220-y)
Supplement: Supplementary file 1 — Additional file 1. Additional details on methods. [file 13613_2023_1220_MOESM1_ESM.docx]

**Inhaled drug delivery: A randomized study in intubated patients with healthy lungs**

Jonathan Dugernier, P.T., Ph.D., Deborah Le Pennec, Guillaume Maerckx, P.T., Laurine Allimonnier, Michel Hesse, Ph.D., Diego Castanares Zapatero, M.D., Ph.D., Virginie Depoortere, NMT., Laurent Vecellio, Ph.D., Gregory Reychler, P.T., Ph.D., Jean-Bernard Michotte, P.T., Ph.D., Pierre Goffette, M.D., Ph.D., Marie-Agnes Docquier, M.D., Ph.D., Christian Raftopoulos, M.D., Ph.D., François Jamar, M.D., Ph.D., Pierre-François Laterre, M.D., Stephan Ehrmann, M.D., Ph.D., and Xavier Wittebole, M.D.

**Additional file 1 – Additional details on methods**

**Patient selection**

Patients were included if they had an healthy lung function defined as a ratio of the forced expiratory volume at 1 second to the forced vital capacity superior to 70%. Written informed consent was obtained from all participants before the surgery. Pulmonary function testing was performed during the preoperative phase according to the American Thoracic Society guidelines using a MicroLoop spirometer (CareFusion, San Diego, CA) (1).

**Image acquisition and deposition analysis**

All static anterior acquisitions for pulmonary and extrapulmonary deposition analysis lasted 2 minutes. The outlining of the lungs necessary to design the lung regions of interest (ROI) required a 6-minutes duration transmission scan using a flood-field source of ^99m^Tc. A dynamic acquisition (i.e. succession of 2-min anterior planar imaging of the thorax) was performed during the whole duration of the nebulization.

Delineation of lung outlines, regions of interest and activity quantification were performed using a home-made plug-in to ImageJ software (RasbandWS, http://imagej.nih.gov/ij/, 1997–2022, Bethesda, MD) according to international recommendations (E2). All data were anonymized by a code number. Data analysis was blinded, i.e. performed by a physician of the Nuclear Medicine department without knowledge of the allocated group.

Right and left lung deposition were separately measured using a rectangular ROI fitted to the lung outlines defined by an isocontour set at 36% of the maximum pixel on the ^99m^Tc flood-field source transmission scan (**see Additional file 2**). Pulmonary deposition was derived from the quantification of counts included in these ROIs. A right to left lung deposition ratio was calculated to compare both lungs deposition. A penetration index was calculated to evaluate the penetration of aerosol particles from an inner to an outer rectangular lung region designed on the ^99m^Tc flood scan and copied on the ^99m^Tc-DTPA scan (**see Additional file 2**). The penetration index was calculated as the outer-to-inner lung region ratio (O/I) from the ^99m^Tc-DTPA acquisition normalized to the O/I ratio from the ^99m^Tc flood-field acquisition (2). A complementary analysis was performed to correct lung deposition data for drug trickling from the tracheal area after the end of the nebulization if an increase of activity superior to 10% was measured in lung ROI, i.e. superior to the Poisson error inherent to planar scintigraphic analysis. Extrapulmonary deposition was determined by the total count measured in the nebulizer reservoir minus whole lung count. Deposition analysis within the endotracheal tube, the trachea and the main bronchi was determined by the total extrapulmonary deposition minus the total count in the ventilation circuit and retained the nebulizer reservoir. They were evaluated together due to the difficult differentiation of the deposition in the distal part of the endotracheal tube and the trachea. The activity in the ventilator circuit was measured placing each component directly on the camera head. A heat and moisture exchanger (HME) filter (Hygrovent S, Flexicare Medical Ltd, Mountain Ash, UK) was placed on the expiratory limb during nebulization to measure the exhaled dose and to avoid radioactive particles liberation. Radiolabeled deposition on each component of the ventilation circuit and the expiratory filter was quantified using a ROI fitted to their size. The activity measured in each ROI was corrected for background using mean pixel count of a region outside of the radiolabeled area. The corrected activity (C) in each ROI was calculated using this formula:

$$C=C_{ROI} - \left( \frac{C_{Bg}}{A_{Bg}} x A_{ROI} \right)$$

, where C_ROI or Bg_ represents the total counts measured in the radiolabeled (ROI) or in the background region (Bg) and A_ROI or Bg_, is the corresponding region surface.

The activity was also corrected for decay of the radioisotope using a time correction factor (TCF):

$$\mathrm{TCF}= e^{\frac{0,693 x t}{360}}$$

, where t (minutes) was the time between the acquisition of the filled nebulizer and each image of ^99m^Tc deposition. The resulting count rate corrected for background and attenuation was multiplied by this TCF.

Chest tissue attenuation of gamma rays should be corrected using an attenuation correction factor (ACF). The ACF for lungs was calculated using the flood-field source method (^99m^Tc flood-field source) for each patient. We used this formula:

$$\mathrm{ACF}= \sqrt{\frac{C_{0}}{C_{1}}}$$

, where C_0_ and C_1_ are the counts in a ROI from the flood-field source of ^99m^Tc without and with the subject present, respectively.

The potential radioactivity on the expiratory valve of the ventilator and the room (ambient and surface contamination) was measured using a portable radiation detector (Mini 900 Scintillation Monitor, Thermo Fisher Scientific ™, Waltham, MA).

**References**

1. Miller MR, Hankinson J, Brusasco V, Burgos F, Casaburi R, Coates A, et al. Standardisation of spirometry. Eur Respir J 2005;26:319–338.

2. Newman S, Bennett WD, Biddiscombe M, Devadason SG, Dolovich MB, Fleming J, et al. Standardization of Techniques for Using Planar (2D) Imaging for Aerosol Deposition Assessment of Orally Inhaled Products. J Aerosol Med Pulm Drug Deliv 2012;25:S-10.
