# Supplementary figures and images for: Inhaled drug delivery: a randomized study in intubated patients with healthy lungs
Source: Ann Intensive Care. 2023 Dec 11;13:125. doi: 10.1186/s13613-023-01220-y (PMC10710976; doi:10.1186/s13613-023-01220-y)

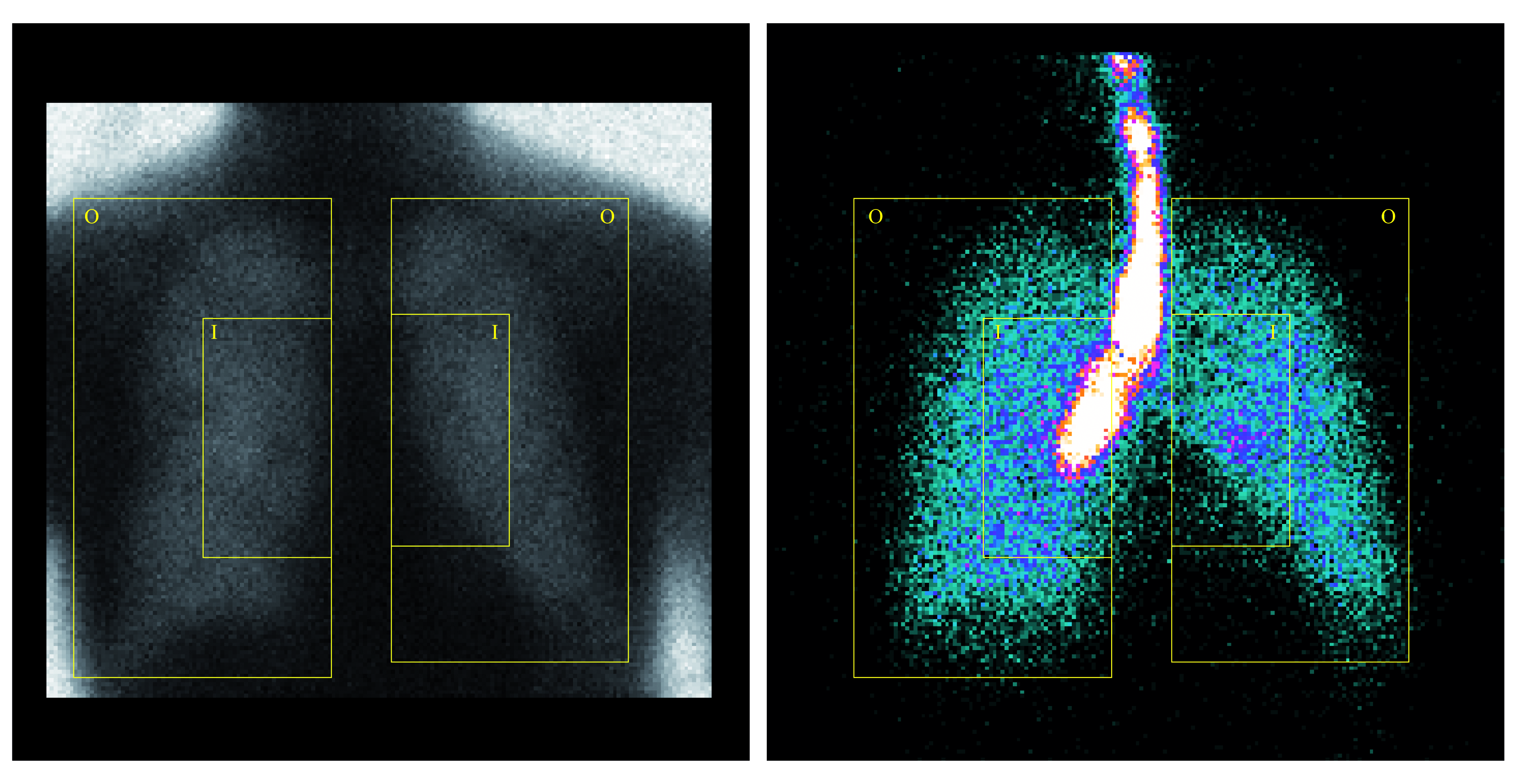

Supplement: Supplementary file 2 — Additional file 2. Definition of lung regions to assess aerosol distribution and penetration in the right and the left lung during invasive mechanical ventilation. (A) Both lungs were divided into inner (I) and outer (O) region of interest using the 99mTc flood ventilation scan. The limits of the rectangular outer lung region were defined as the 36% isocount contour of peak counts in the lung field without artefacts (i.e. “islands” or “peninsulas” inside or outside of the lung field). The inner region was automatically designed as half of the width of the outer region and one-half of its height. (B) Regions of interest were copied on the 99mTc-DTPA deposition scan to quantify the inhaled radiolabeled drug deposition. [file 13613_2023_1220_MOESM2_ESM.tif]

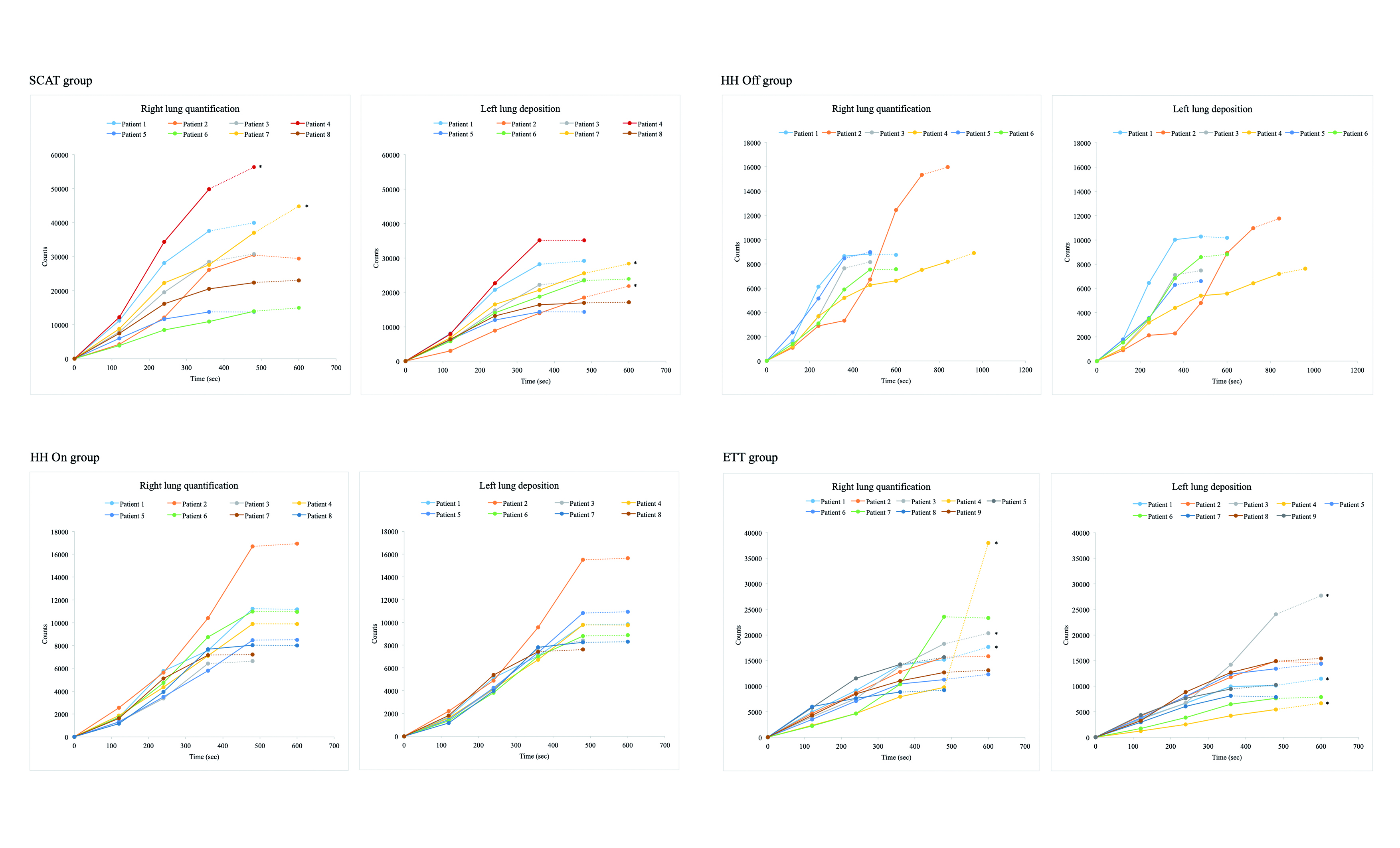

Supplement: Supplementary file 4 — Additional file 4. Evolution of the activity deposited inside the right and the left lung regions was measured throughout the dynamic acquisition during the nebulization (plain line) and after the nebulization (dashed line). All data points were normalized to 120-s acquisitions. Three patients from the SCAT group (asterisk) and three patients from the ETT group (asterisk) had an increase of counts superior to 10% between the last dynamic acquisition and the acquisition for lung deposition, a difference considered significant as it exceeds the Poisson error inherent to planar scintigraphy analysis. This increase in activity after the nebulization was linked to drug trickling from the endotracheal tube and the tracheal area to the lungs. This phenomenon probably happened even during the nebulization, as indicated by an exponential increase of counts, but cannot be quantified. ETT, endotracheal tube; HH, heated humidifier; SCAT, specific ventilator circuit for aerosol therapy. [file 13613_2023_1220_MOESM4_ESM.tif]
