# Supplementary material for: Inhaled drug delivery: a randomized study in intubated patients with healthy lungs
Source: Ann Intensive Care. 2023 Dec 11;13:125. doi: 10.1186/s13613-023-01220-y (PMC10710976; doi:10.1186/s13613-023-01220-y)
Supplement: Supplementary file 3 — Additional file 3. Aerodynamic characteristics of aerosol particles. [file 13613_2023_1220_MOESM3_ESM.docx]

**Inhaled drug delivery: A randomized in vivo study in intubated patients with healthy lungs**

Jonathan Dugernier, P.T., Ph.D., Deborah Le Pennec, Guillaume Maerckx, P.T., Laurine Allimonnier, Michel Hesse, Ph.D., Diego Castanares Zapatero, M.D., Ph.D., Virginie Depoortere, NMT., Laurent Vecellio, Ph.D., Gregory Reychler, P.T., Ph.D., Jean-Bernard Michotte, P.T., Ph.D., Pierre Goffette, M.D., Ph.D., Marie-Agnes Docquier, M.D., Ph.D., Christian Raftopoulos, M.D., Ph.D., François Jamar, M.D., Ph.D., Pierre-François Laterre, M.D., Stephan Ehrmann, M.D., Ph.D., and Xavier Wittebole, M.D.

**Additional file 3**

**Table.** Aerodynamic characteristics of aerosol particles

|  | **SCAT group**  **(n =8)** | **HH Off group**  **(n = 6)** | **HH On group**  **(n = 8)** | **ETT group**  **(n = 9)** |
| --- | --- | --- | --- | --- |
| MMAD (μm) at the outlet of the nebulizer | 3.8 ± 0.4 | 3.7 ± 0.2 | 3.8 ± 0.2 | 4.1 ± 0.4 |
| MMAD (μm) at the distal tip of the ETT | 2.7 ± 1 | 2.6 ± 0.6 | 2.2 ± 0.65 | 2.9 ± 0.9 |
| FPF (% < 5 μm) at the distal tip of the ETT | 79.5 ± 12 | 83.6 ± 8.5 | 87.8 ± 6.5 | 76.1 ± 9.9 |

Data expressed as mean ± SD.

ETT, endotracheal tube; FPF, fine particle fraction; HH, heated humidifier; MMAD, mass median aerodynamic diameter; SCAT, specific ventilation circuit for aerosol therapy.
