# Supplementary material for: Inhaled drug delivery: a randomized study in intubated patients with healthy lungs
Source: Ann Intensive Care. 2023 Dec 11;13:125. doi: 10.1186/s13613-023-01220-y (PMC10710976; doi:10.1186/s13613-023-01220-y)
Supplement: Supplementary file 5 — Additional file 5. Aerosol deposition after correction for drug trickling from the endotracheal tube to the lungs after the nebulization. [file 13613_2023_1220_MOESM5_ESM.docx]

**Inhaled drug delivery: A randomized study in intubated patients with healthy lungs**

Jonathan Dugernier, P.T., Ph.D., Deborah Le Pennec, Guillaume Maerckx, P.T., Laurine Allimonnier, Michel Hesse, Ph.D., Diego Castanares Zapatero, M.D., Ph.D., Virginie Depoortere, NMT., Laurent Vecellio, Ph.D., Gregory Reychler, P.T., Ph.D., Jean-Bernard Michotte, P.T., Ph.D., Pierre Goffette, M.D., Ph.D., Marie-Agnes Docquier, M.D., Ph.D., Christian Raftopoulos, M.D., Ph.D., François Jamar, M.D., Ph.D., Pierre-François Laterre, M.D., Stephan Ehrmann, M.D., Ph.D., and Xavier Wittebole, M.D.

**Additional file 5**

**Table.** Aerosol deposition after correction for drug trickling from the endotracheal tube to the lungs after the nebulization

|  | **SCAT group**  **(n =8)** | **HH Off group**  **(n = 6)** | **HH On group**  **(n = 8)** | **ETT group**  **(n = 9)** |
| --- | --- | --- | --- | --- |
| **Pulmonary deposition (%)** | **19.2 (13.6-23.6)*†‡** | **6.2 (5.4-7.3)§** | **6.9 (6.6-7.4)§** | **8.7 (7.2-9.4)§** |
| Right lung | 10.5 (8.1-13.3)*†‡ | 3.1 (2.7-3.7)§ | 3.5 (3.0-3.9)§ | 4.3 (3.8-5.8)§ |
| - Inner region | 5.2 (3.6-6.0)*†‡ | 1.2 (1.0-1.5)§ | 1.3 (1.2-1.5)§ | 1.8 (1.5-2.9)§ |
| - Outer region | 5.4 (4.0-7.7)*†‡ | 1.8 (1.7-2.3)§ | 2.2 (1.9-2.5)§ | 2.5 (2.1-2.8)§ |
| - Penetration index | 0.44 ± 0.12 (27)*† | 0.66 ± 0.10 (15)§ | 0.67 ± 0.07 (10)§ | 0.53 ± 0.21 (40) |
| Left lung | 8.6 (6.0-10.2)*†‡ | 3.1 (2.6-3.7)§ | 3.5 (3.1-4.1)§ | 3.8 (2.6-4.8)§ |
| - Inner region | 3.4 (2.6-4.0)*†‡ | 1.1 (0.9-1.3)§ | 1.3 (1.2-1.4)§ | 1.4 (1.0-2.0)§ |
| - Outer region | 4.9 (3.6-6.3)*†‡ | 2.0 (1.7-2.3)§ | 2.1 (1.9-2.7)§ | 2.3 (1.6-2.8)§ |
| - Penetration index | 0.57 ± 0.15 (26) | 0.73 ± 0.10 (14) | 0.69 ± 0.11 (16) | 0.59 ± 0.12 (20) |
| Right/Left lung ratio | 1.30 ± 0.23 (17) | 1.04 ± 0.17 (16) | 1.01 ± 0.19 (19) | 1.34 ± 0.65 (48) |
| **Extrapulm. deposition (%)** | **80.8 (76.4-86.4)*†‡** | **93.8 (92.7-94.6)§** | **93.1 (92.5-93.4)§** | **91.3 (90.6-92.8)§** |
| ETT and Tracheal area | 23.9 (20.3-28.8)*† | 11.9 (11.1-12.1)‡§ | 7.4 (6.7-8.6)‡§ | 19.2 (17.0-24.6)*† |

Aerosolized drug trickling from the endotracheal tube and the trachea to the lungs was observed for three patients of the SCAT group and three patients of the ETT group. Quantification was only corrected for drug trickling after the nebulization only, not during the nebulization. Data are expressed as mean ± SD (coefficient of variation, %) or median (25-75% IQR) percentage of the nominal dose. ETT, endotracheal tube; HH, heated humidifier; SCAT, specific ventilator circuit for aerosol therapy.

* p < 0.05 vs HH Off group

† p < 0.05 vs HH On group

‡ p < 0.05 vs ETT group

§ p < 0.05 vs SAT group.
