# Supplementary material for: Inhaled drug delivery: a randomized study in intubated patients with healthy lungs
Source: Ann Intensive Care. 2023 Dec 11;13:125. doi: 10.1186/s13613-023-01220-y (PMC10710976; doi:10.1186/s13613-023-01220-y)
Supplement: Supplementary file 6 — Additional file 6. In vitro analysis of aerosol deposition in invasive mechanical ventilation. [file 13613_2023_1220_MOESM6_ESM.docx]

**Inhaled drug delivery: A randomized study in intubated patients with healthy lungs**

Jonathan Dugernier, P.T., Ph.D., Deborah Le Pennec, Guillaume Maerckx, P.T., Laurine Allimonnier, Michel Hesse, Ph.D., Diego Castanares Zapatero, M.D., Ph.D., Virginie Depoortere, NMT., Laurent Vecellio, Ph.D., Gregory Reychler, P.T., Ph.D., Jean-Bernard Michotte, P.T., Ph.D., Pierre Goffette, M.D., Ph.D., Marie-Agnes Docquier, M.D., Ph.D., Christian Raftopoulos, M.D., Ph.D., François Jamar, M.D., Ph.D., Pierre-François Laterre, M.D., Stephan Ehrmann, M.D., Ph.D., and Xavier Wittebole, M.D.

**Additional file 6**

**Table.** *In vitro* analysis of aerosol deposition in invasive mechanical ventilation

|  | **SCAT group**  **(n =8)** | **HH Off group**  **(n = 6)** | **HH On group**  **(n = 8)** | **ETT group**  **(n = 9)** | **Overall Pearson correlation with *in vivo* data** | |
| --- | --- | --- | --- | --- | --- | --- |
|  |  |  |  |  | **r** | ***p-value*** |
| **Inhaled fraction (%)** | 32.5 (27.1-33.4)*† | 15.4 (11.3-17.3)‡§ | 12.4 (9.9-22.7)‡§ | 21.2 (19.7-31.8)*† | **0.768** | **<0.001** |
| **Extrapulmonary**  **deposition (%)** | 69.4 (66.6-74.6)*† | 85.5 (81.5-89.4)‡§ | 88.2 (78.5-89.5)‡§ | 77.5 (69.7-79.8)*† | **0.768** | **<0.001** |
| ETT | 8.2 (4.7-11.7)*†‡ | 2.4 (1.4-3.0)‡§ | 0.4 (0.2-1.0)‡§ | 12.2 (9.7-16.8)*†§ | **0.732** | **<0.001** |
| Ventilation circuit | 56.9 (47.5-61.4)*†‡ | 74.7 (71.8-78.1)‡§ | 79.2 (69.3-81.4)‡§ | 55.7 (50.8-60.4)*†§ | **0.817** | **<0.001** |
| Inspiratory circuit | 41.5 ± 6.3 (15)*†‡ | 69.2 ± 4.5 (6)‡§ | 70 ± 7.6 (11)‡§ | 19.1 ± 5.6 (29)*†§ | **0.959** | **<0.001** |
| - Inspiratory limb | 22.7 ± 10.2 (45) | 24.4 ± 4.4 (18) | 25.5 ± 9.0 (35) | / | 0.045 | 0.843 |
| - Humid. chamber | / | 35.7 ± 2.3 (6) | 33.4 ± 4.6 (14) | / | **-0.536** | **0.048** |
| - Nebulizer T-piece | 18.9 ± 10.4 (55)* | 9.1 ± 1.8 (20)‡§ | 11.1 ± 3.0 (27) | 19.1 ± 5.6 (29)* | 0.157 | 0.400 |
| Expiratory circuit | 10.4 (9.1-19.2)*†‡ | 5.5 (4.7-6.4)‡§ | 5.9 (4.7-8.3)‡§ | 36.4 (32.9-39.8)*†§ | **0.952** | **<0.001** |
| Nebulizer retention | 6.5 ± 1.5 (23) | 8.3 ± 1.6 (19) | 8.2 ± 1.4 (17) | 6.9 ± 1.7 (25) | -0.253 | 0.169 |

The thirty-one patients from the *in vivo* analysis were replicated *in vitro*. Aerosol deposition was quantify in each component of the ventilation circuit, the endotracheal tube and the filter at the distal tip of the endotracheal tube, representing the amount of drug reaching the respiratory tract, i.e. the inhaled fraction. Data are expressed as mean ± SD (coefficient of variation, %) or median (25-75% IQR) percentage of the nominal dose. ETT, endotracheal tube; HH, heated humidifier; SCAT, specific ventilator circuit for aerosol therapy.

Inter-group comparison:* p < 0.05 vs HH Off group; † p < 0.05 vs HH On group; ‡ p < 0.05 vs ETT group; § p < 0.05 vs SAT group.
